# Batched Bayesian Optimization for Drug Design in Noisy Environments

**DOI:** 10.1021/acs.jcim.2c00602

**Published:** 2022-08-31

**Authors:** Hugo Bellamy, Abbi Abdel Rehim, Oghenejokpeme I. Orhobor, Ross King

**Affiliations:** Department of Chemical Engineering and Biotechnology, University of Cambridge, Philippa Fawcett Drive, Cambridge CB3 0AS, UK

## Abstract

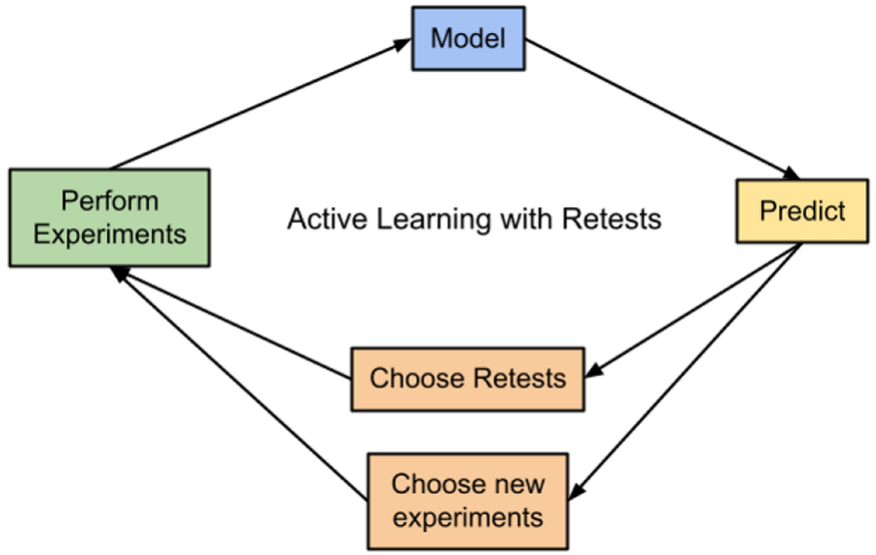

The early stages of the drug design process involve identifying compounds with suitable bioactivities via noisy assays. As databases of possible drugs are often very large, assays can only be performed on a subset of the candidates. Selecting which assays to perform is best done within an active learning process, such as batched Bayesian optimization, and aims to reduce the number of assays that must be performed. We compare how noise affects different batched Bayesian optimization techniques and introduce a retest policy to mitigate the effect of noise. Our experiments show that batched Bayesian optimization remains effective, even when large amounts of noise are present, and that the retest policy enables more active compounds to be identified in the same number of experiments.

## Introduction

The early stages of the drug discovery process involve identifying suitable compounds in assays. These are prone to experimental random error, caused by random variations in natural processes.^1^ This study focuses on how to select which assays to perform to quickly identify suitable compounds, given the presence of noise. This is an important problem, as reducing the number of experiments would help reduce the large costs and time scales currently involved in drug design; typically, new drugs cost upward of $2.5 billion, and the process takes approximately 10 years to complete.^2^

The standard approach to this problem is to use virtual screening techniques, most commonly quantitative structure–activity relationship (QSAR) models, to identify potential drug candidates from large databases of chemicals.^3,4^ A QSAR model links a chemical descriptor to an activity value: the chemical descriptor can be either a two-dimensional (2D) or three-dimensional (3D) representation of the molecule that provides information about the chemical structure; the activity value will be the property of interest—in drug design this is usually binding affinity to a target protein.^5^ An initial set of known activity values is required with which to train the model, and the accuracy of the model’s predictions will depend on the size and quality of this initial data set.

Active learning provides a system to use these predictions for effective experiment selection; it aims to reduce the amount of data required to achieve a desired outcome by using an algorithm to select future training examples. This technique is particularly applicable to drug design, as chemical activity experiments are expensive to perform, and it has been used to reduce the data requirement across a range of drug design tasks.^6−9^ Batched Bayesian optimization is an active learning method where experiments are performed in batches (reflecting how real drug design experiments are performed^10^), with a surrogate model being used to predict the activity of untested compounds between batches. An acquisition metric then uses the activity predictions to select which compounds will be in the next batch. Batched Bayesian optimization has been shown to be effective across a range of physical tasks including material design^11^ and drug design.^12^ Performing batched Bayesian optimization requires a choice of both surrogate model and acquisition function. Graff et al. compared multiple surrogate models and acquisition functions on a computational docking experiment. They found directed message passing neural networks with an upper confidence bound or greedy acquisition metric to be most effective,^12^ although the effectiveness of any active learning technique will depend on the data set.^13^ Pyzer-Knapp^14^ demonstrated this by testing Bayesian optimization on two different drug discovery data sets. On the smaller, simpler, data set the greedy metric performed the best, with two versions of expected improvement performing only slightly worse. On a larger data set, which presented a more complicated optimization problem by having multiple local maxima, the expected improvement methods had a much better performance than the greedy method, which got stuck in a local maxima and never recovered.

Other approaches to compound screening attempt to balance the need to both find highly active compounds and to explore uncertain areas of the data set. For example, Yang et al. compared four methods of selecting compounds based on there predicted activity and uncertainty.^15^ They found selecting compounds the model had the highest uncertainty on, from the compounds that were in the top 5% of predicted activities, to be an effective active learning method in all cases, allowing for the same performance as the standard approach while using less data.

When noise is present the effectiveness of active learning decreases, and methods that are optimal in noise-free environments can perform poorly when noise is present.^16^ So, it is important to test how active learning performs in noisy environments, both to find techniques that are appropriate for real data sets and to give realistic predictions for the performance of active learning. Methods to minimize the effect of noise in active learning include the development of specific algorithms and repeating experiments. For example, Pickett et al. used a genetic algorithm to select training examples and used retests to help minimize the effects of noisy experiments: each compound was retested a fixed number of times.^17^ They successfully identified compounds in the most active regions of the data set despite having noisy experiments.

Our experiments used batched Bayesian optimization to identify active compounds as quickly as possible. They were performed with different amounts of noise present, both with and without the use of a retest policy. This retest policy selectively chose experiments to repeat, differing from that used by Pickett et al., which retested each compound a fixed number of times. Experiments were performed on a simulated data set, on 288 drug activity data sets from CHEMBL^18,19^ and on two data sets from PubChem. The results show that batched Bayesian optimization remains effective in noisy environments, but the relative performance of different techniques varies depending on both the data set and the amount of noise present. Using the retest policy consistently allowed more actives to be correctly identified when noise was present.

## Methods

### Data Sets

Experiments were performed using a simulated data set, enabling the amount of noise to be controlled, and real QSAR data sets from both PubChem and CHEMBL, demonstrating the usefulness of the approach on real problems. The simulated data set was created using the make regression tool from the scikit learn package (version 1.0.1) and contained 5000 samples with 10 features, 5 of which were informative, and had 1 regression target. The CHEMBL data sets^19^ had the pXC50 value as the activity value for learning, and chemical structures were represented using extended-connectivity fingerprints.^20^ These data sets were obtained directly from Olier et al.^18^ All data sets with over 800 entries were used, giving a total of 288 data sets.

Two data sets were obtained from PubChem, namely, assays AID-1347160 and AID-1893. For these data sets the PubChem activity score was used as the activity value for learning. The structural information used was a 1024-bit, radius 2 Morgan fingerprint calculated using rdkit. Actives for these data sets were defined in the data set, so for these experiments the number of actives differed from 10%. The data set for assay AID-1347160 contained 5444 molecules with 323 actives (5.9% actives), and assay AID-1893 contained 5942 molecules with 117 actives (2.0%). Assay AID-1893 is a percent inhibition data set.

### Noise Generation

To observe the effect of random noise on the active learning process, noise was added to all the data sets. However, in the data sets from CHEMBL and PubChem there will be noise present in the underlying data. Assuming that the noise present in the provided data is normally distributed, the experimental data will have activity values following
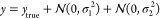
1where *y* is the activity value used in learning, *y*_true_ is the true activity value, σ_1_^2^ is the variance in the noise in the data, and σ_2_^2^ is the variance in the artificially added noise. This equation can be rewritten as^21^
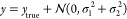
2

The value of σ_1_^2^ will typically be around 0.6^5^ for QSAR data sets. Varying σ_2_^2^ can only show the effect of more noise (with the simulated data σ_1_^2^ = 0 so the effect of noise can be observed directly).

The initial set of random noise was produced using a random seed that changed each run of the experiment. This same set of noise was used for all acquisition functions in a given run to prevent the comparison between acquisition functions being affected by differences in the random generation of noise. Similarly, in experiments when retests were required the noise generation was constant between runs. In experiments noise was added with variance, σ_2_^2^, proportional to the range of *y* values in the data.
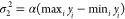
3The values of α used were: 0, 0.05, 0.1, 0.15, 0.2, and 0.25. The average range of the activity values in the CHEMBL data sets was 6, meaning that σ_2_^2^ and σ_1_^2^ are of similar size.

### Active Learning

The active learning process was performed as batched Bayesian optimization, with 100 molecules per batch, which is also used by Graff et al.^12^ A QSAR model was trained using a randomly selected initial batch of 100 molecules; this surrogate model was then used to predict the activity of the remaining molecules. Various acquisition functions were used to rank untested molecules by the estimated utility of performing an experiment on them. The inputs for the acquisition function are model prediction, model uncertainty, and the required activity for a molecule to be considered active; full details are given later in the methods section.^22^ The next batch of experiments was selected by taking the top 100 molecules as ranked by the acquisition function. This is a naive method to select batches: all molecules are selected independently of each other, which can be suboptimal,^23^ but it greatly decreases the computational complexity of the process compared to other batch selection policies.^24^

After a batch is selected the activity readings for the new molecules are added to the data set. If retests were being used, the molecules to be retested were identified. Each retest would mean one less new molecule in the next batch, to keep the total number of experiments at 100 per batch. With the new data, a new QSAR model was generated to give a new set of predicted activities, to be used to rank molecules via the acquisition functions. The next batch was then found by combining the molecules identified to be retested with the top ranked molecules to make a total batch of 100 (or just the top 100 if not using retests). This process continues until the total number of measurements performed is greater than half the total number of entries in the data set (e.g., if the data set contains 5100 entries, 25 active learning batches will be performed after the initially selected random batch). See the experimental design subsection at the end of the methods section for details on which experiments were performed.

### Acquisition Functions

The acquisition functions tested were
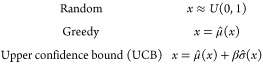

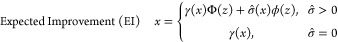

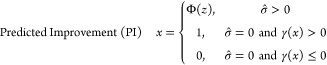
where
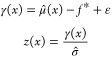
μ̂(*x*) and σ̂(*x*) are the predicted mean and uncertainty at point *x*, respectively. Φ and ϕ are the cumulative distribution function and the probability density function of the standard normal distribution, respectively, and f* is the target objective function value. For the experiments in this paper β = 2 and ε = 0.01 were used. These metrics are the same as those used by Graff et al.^12^

### Surrogate Models

The QSAR models were made using random forest regression, implemented in python using the sckit learn package (version 1.0.1). Each forest used 100 trees, and the default values are used for the remaining parameters: all the variables are considered at each split, the squared error criterion is used to measure the quality of the split, and only one sample is required to be a leaf node. Random forest models were chosen, as they have been shown to provide good performance on QSAR problems.^18,25^

### Retest Policy

To try and reduce the number of active molecules that are incorrectly labeled as inactive a retest policy was added. If a molecule was predicted to be above the active threshold, but the measured value was below this threshold, it was retested. A retest is subject to the same amount of random noise as the original test. Both this new activity value and the original are used in the training set. This is because there is no reason to believe either of the measurements to be more valid than the other, as both have been randomly sampled from the same distribution. For each molecule that is being retested one less new molecule is added in the next batch; this keeps the number of measurements in the data set consistent, allowing for a comparison between results.

### Hit Detection

The objective of the active learning process in this investigation was to quickly detect active compounds. Actives were defined as molecules within the top 10% of the entire data set. An active compound was found after it had been recommended by the active learner and added to the training set. However, because of the added noise, when the active compound is added to the data set its measured activity value may be below the threshold for it to be considered active. A compound that is active and has been recorded as active is referred to as a “true active”.

### Experimental Design

For each data set experiments are performed both with and without retests. They are done as follows.

#### Without Retests

1. 100 sample random initial batch selected as a training set and used to train a model.
2. A batch of 100 samples is selected using the acquisition metric with the model predictions, the batch is added to the training set, and the model is retrained.
3. The number of actives and true actives present in the training set are recorded.
4. Steps 2 & 3 are repeated until the total number of entries in the training set is greater than half the total entries in the data set.

This process is referred to as a run and is done 10 times for each combination of acquisition metric and noise level, with the noise generation being different each time.

#### With Retests

1. Initially the list of molecules to be retested is empty and so has a length of *n* = 0.
2. 100 sample random initial batch selected as a training set and used to train a model.
3. A batch of 100 – *n* samples is selected using the acquisition metric with the model predictions.
4. This is combined with list of molecules to be retested to get the full batch.
5. Measurements are obtained for samples in the batch and used to determine if they should be retested, this information is stored as the list of molecules to be retested of length *n*.
6. The batch is added to the training set, and the model is retrained.
7. The number of actives and true actives present in the training set is recorded.
8. Steps 3–7 are repeated until the total number of entries in the training set is greater than half the total entries in the data set; note that, due to retests, samples may be repeated in the training set.

Again, this process is referred to as a run and is done 10 times for each combination of acquisition metric and noise level, with the noise generation being different each time.

## Results

### Simulated Data Sets

**Figure 1:**
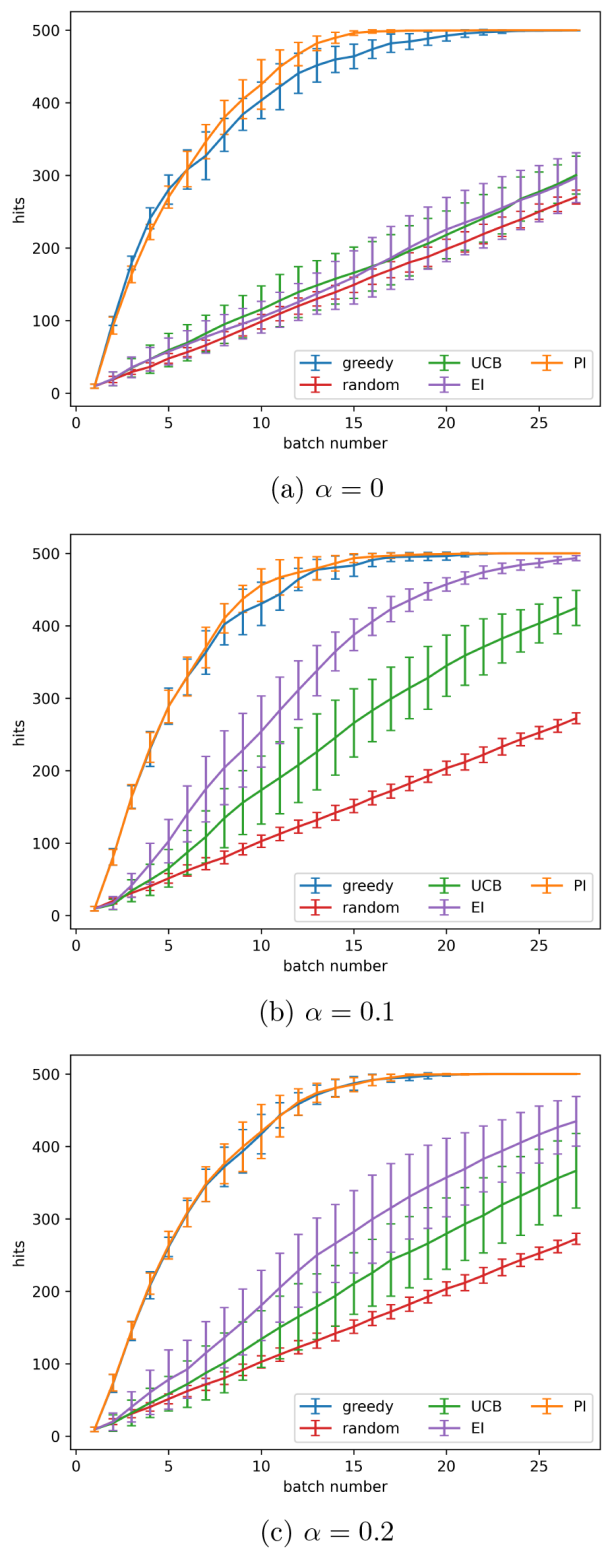
Active learning performance with different amounts of noise present using the simulated data set. Noise values (σ_2_^2^) are found using eq 3 with the indicated values for α. Results shown are the mean over 10 runs; error bars show the standard deviation. Acquisition metrics: greedy, random, UCB - upper confidence bound, EI - expected improvement, PI - predicted improvement.

**Figure 2:**
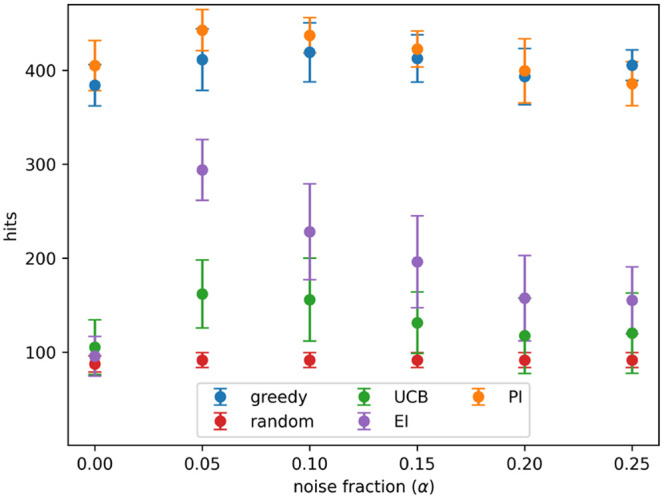
Hits found after 8 active learning batches for each acquisition metric on the simulated data set with different levels of noise. Noise added using eq 3 and the indicated values for α. Results show the mean of 10 runs, and the error bars indicate the standard deviation. Acquisition metrics: greedy, random, UCB - upper confidence bound, EI - expected improvement, PI - predicted improvement.

The active learning process was performed, without retests, on the simulated data set as described in the methods section. Noise was added to the data set with variance given by eq 3, using α values of 0, 0.05, 0.1, 0.15, 0.2, and 0.25. This process enabled a comparison of the acquisition metrics. Figure 1 shows how hits found varied as batch number increased for the different acquisition metrics using α values of 0, 0.1, and 0.2. Figure 2 shows the number of hits found after 8 active learning batches for each acquisition metric at each noise level (α value) tested. At all noise levels tested batched Bayesian optimization, using the greedy and PI acquisition metrics, outperformed random selection. The UCB and EI acquisition metrics performed poorly: they found fewer hits than PI and greedy at all noise levels and performed similarly to a random search at high noise levels.

**Figure 3:**
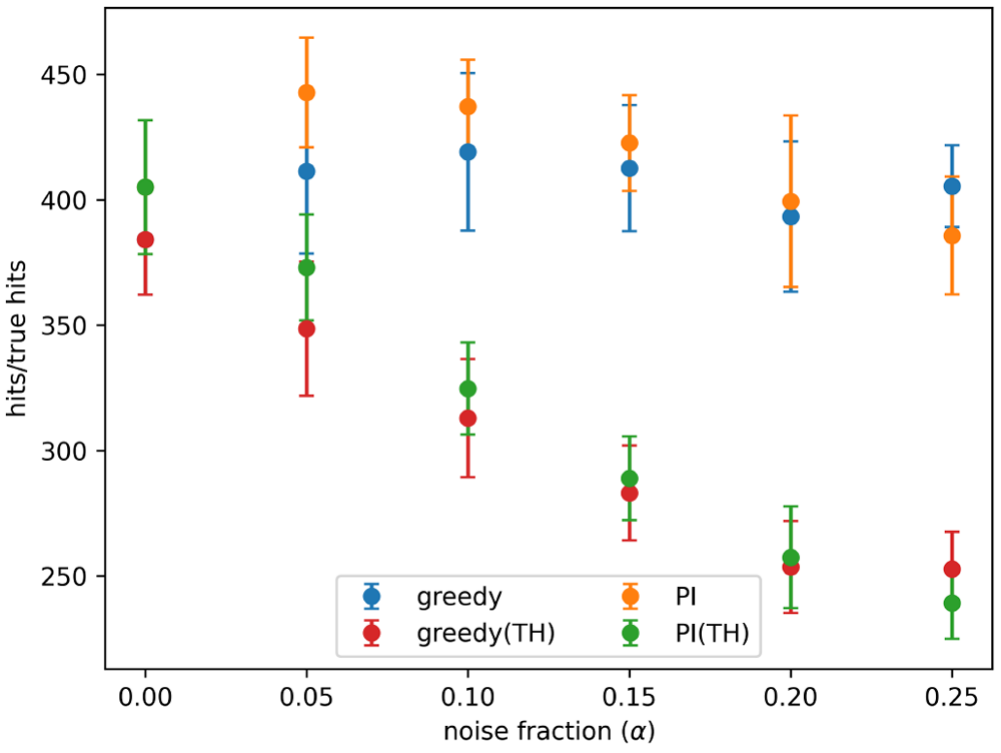
Hits and true hits found after 8 active learning batches for the predicted improvement (PI) and greedy acquisition metrics. Noise added using eq 3 and the indicated values for α. Results show the mean of 10 runs, and the error bars indicate the standard deviation.

Figure 3 shows the number of hits and true hits detected after 8 active learning batches, for the greedy and PI acquisition metrics, at all noise levels tested. The rate of detection of true hits decreases rapidly as noise increases for both acquisition metrics. A hit is recorded when an active sample is added to the training set, but for a sample to be a true hit it must be added to the training set and also have an appropriately high measured activity value. So, the difference between the detection of hits and true hits is due to random noise causing active samples to sometimes have low measured activity values.

**Figure 4:**
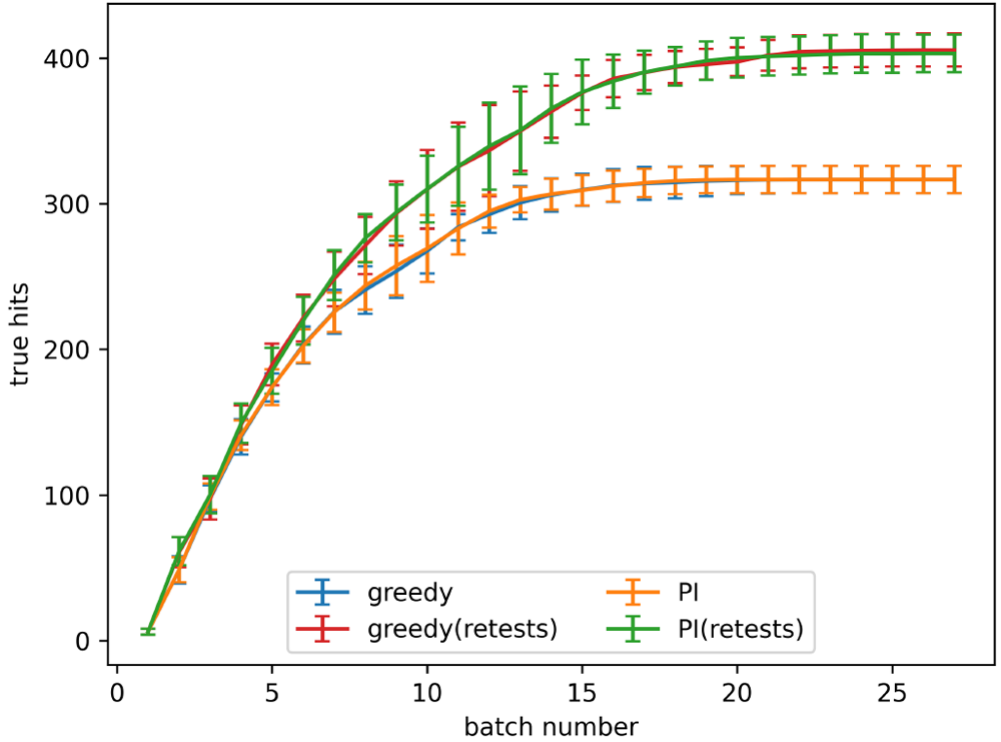
True hits found using active learning, both with and without retests, on the simulated data set for the predicted improvement (PI) and greedy acquisition metrics. Noise added using α = 0.2 in eq 3. The graph shows the mean of 10 runs, and the error bars indicate the standard deviation.

**Figure 5:**
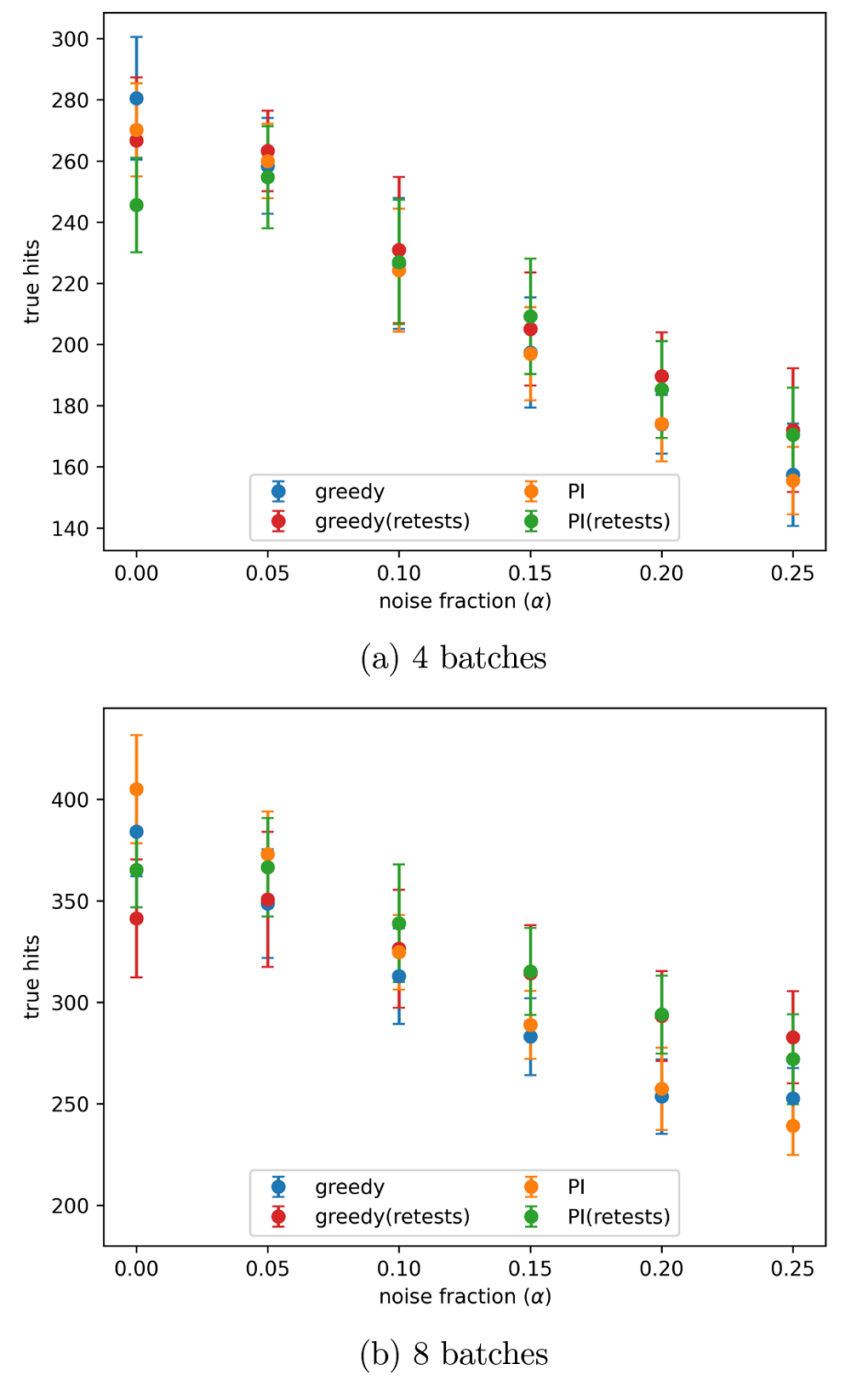
Number of true hits found after the indicated number of active learning batches, with different levels of artificial noise in the data, both with and without retests. Noise added using the indicated α values in eq 3. Results are the mean of 10 runs with the error bars showing the standard deviation. The acquisition metrics used were greedy and predicted improvement (PI).

The retest policy described in the methods section was implemented with the goal of finding more true hits. The rate of true hit acquisition both with and without retests, with a noise level given by α = 0.2 in eq 3, is shown in Figure 4. The number of true hits found after 4 and 8 batches for all noise levels tested is shown in Figure 5a,b, respectively. When no noise is present using retests causes fewer true hits to be found. When noise is present, active learning processes with a small number of batches find a similar number of hits both with and without retests. As the number of batches increases using retests becomes more beneficial.

### CHEMBL Data Sets

**Figure 6:**
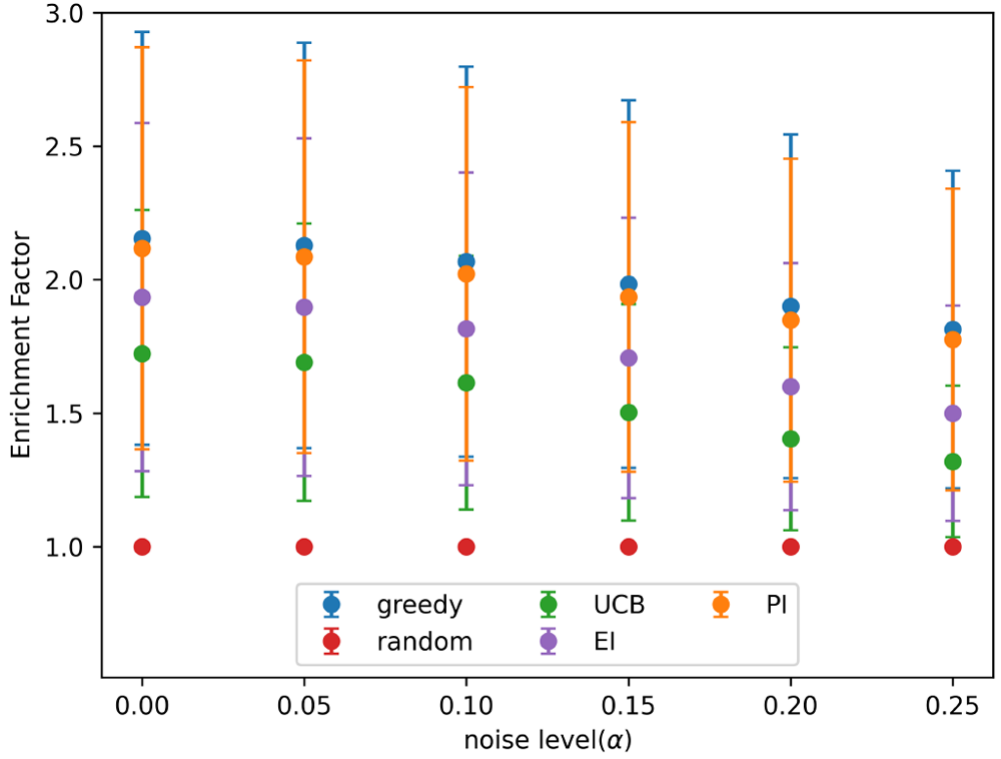
Mean enrichment factor for each acquisition function, at different noise levels, after approximately 25% of the data set had been added in batched Bayesian optimization. Noise added using the indicated α values in eq 3. The graph shows the mean over all data sets tested, and the error bars indicate the standard deviation. Acquisition metrics: greedy, random, UCB - upper confidence bound, EI - expected improvement, PI - predicted improvement.

The batched Bayesian optimization process was then tested on 288 drug activity data sets from CHEMBL. Following Graff et al. an enrichment factor was defined as the ratio of hits (or true hits) found by an acquisition metric to the hits found by random selection. Figure 6 shows the mean enrichment factor, at each noise level tested, for each acquisition metric with error bars showing the standard deviation in results. These results are after approximately 25% of the data set had been used in batched Baysian optimization and noise had been added to the data using eq 3 and the indicated α values. On the real data sets the performance of the acquisition metrics was more similar. All metrics outperformed random selection, and at all noise levels the greedy and PI metrics found the most hits, with the difference increasing as the amount of noise increased.

**Figure 7:**
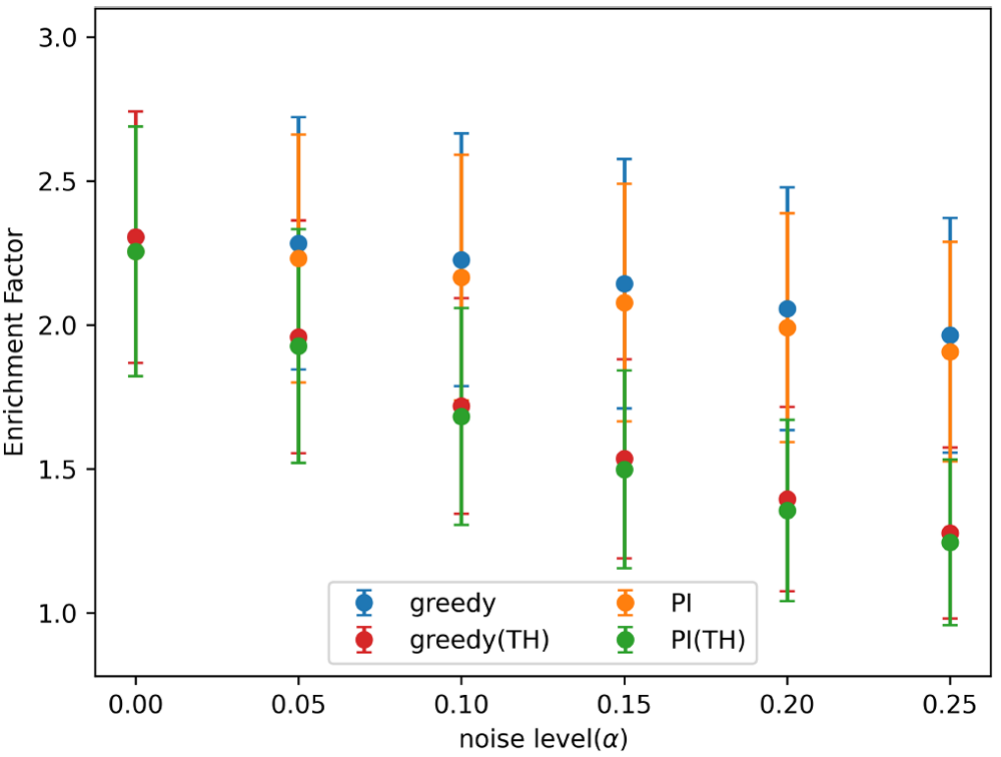
Mean enrichment factor for finding both hits and true hits (true hits indicated by TH), at different noise levels, after approximately 25% of the data set had been added in batched Bayesian optimization. Noise added using the indicated α values in eq 3. The graph shows the mean over all data sets tested, and the error bars indicate the standard deviation. Acquisition metrics: greedy, random, UCB - upper confidence bound, EI - expected improvement, PI - predicted improvement.

Figure 7 shows the enrichment factor for finding both hits and true hits with the greedy and PI acquisition metrics, at different levels of artificial noise, after approximately 25% of the data set had been added to the training set in batched Bayesian optimization. Results are shown at all noise levels tested, found using eq 3 and the α values given. The rate of detection of true hits drops off rapidly for both acquisition metrics.

**Figure 8:**
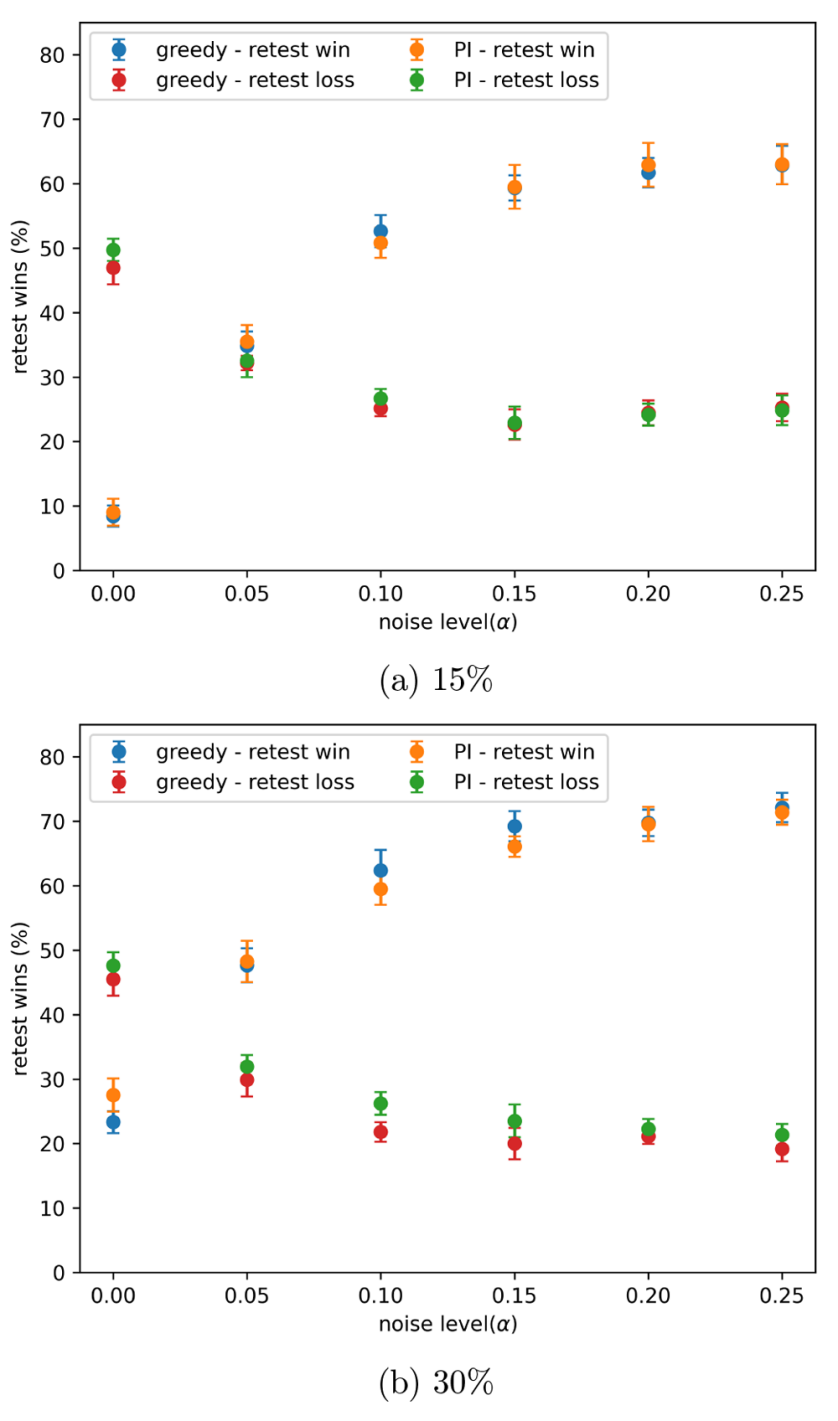
Percentage of data sets using retests finds more true hits than not using retests, with different amounts of noise present, after approximately the indicated percentage of the data set has been added in batched Bayesian optimization. Noise added using the indicated α values in eq 3. Drawn data sets are not shown. Results show the mean of 10 runs, and the error bars indicate the standard deviation.

The restest policy was used to try and find more true hits. The percentage of data sets for which using the retest policy found more true hits than no retests is shown in Figure 8. The results show the mean of 10 runs, and the error bars indicate the standard deviation. The drawn data sets (those that both with and without retests found the same number of true hits) are not shown, so the results on the graph do not add up to 100%. Results are shown for approximately 15% and 30% of the data set being added in Figure 8a,b, respectively. When no noise is present retests are not beneficial; as noise increases retests quickly become more favorable. When more experiments are performed (more of the data set is added), retests win more often. The effectiveness of the retest policy is similar for both the greedy and PI acquisition metrics.

### PubChem Data Sets

**Figure 9:**
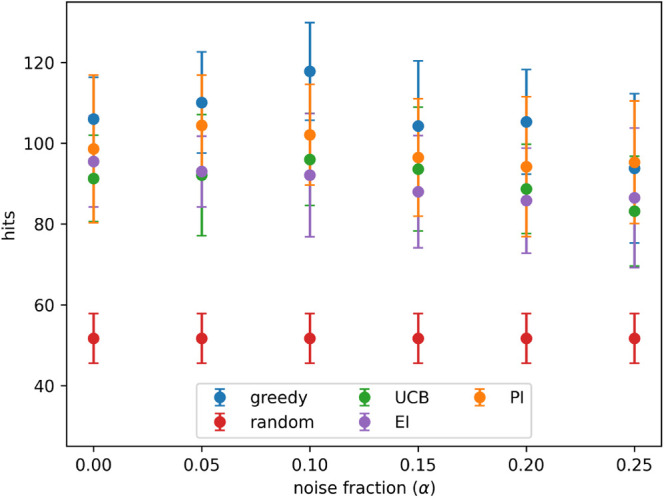
Hits found after 8 active learning batches for each acquisition metric on the PubChem 1347160 data set with different levels of noise. Noise added using eq 3 and the indicated values for α. Results show the mean of 10 runs, and the error bars indicate the standard deviation. Acquisition metrics: greedy, random, UCB - upper confidence bound, EI - expected improvement, PI - predicted improvement.

**Figure 10:**
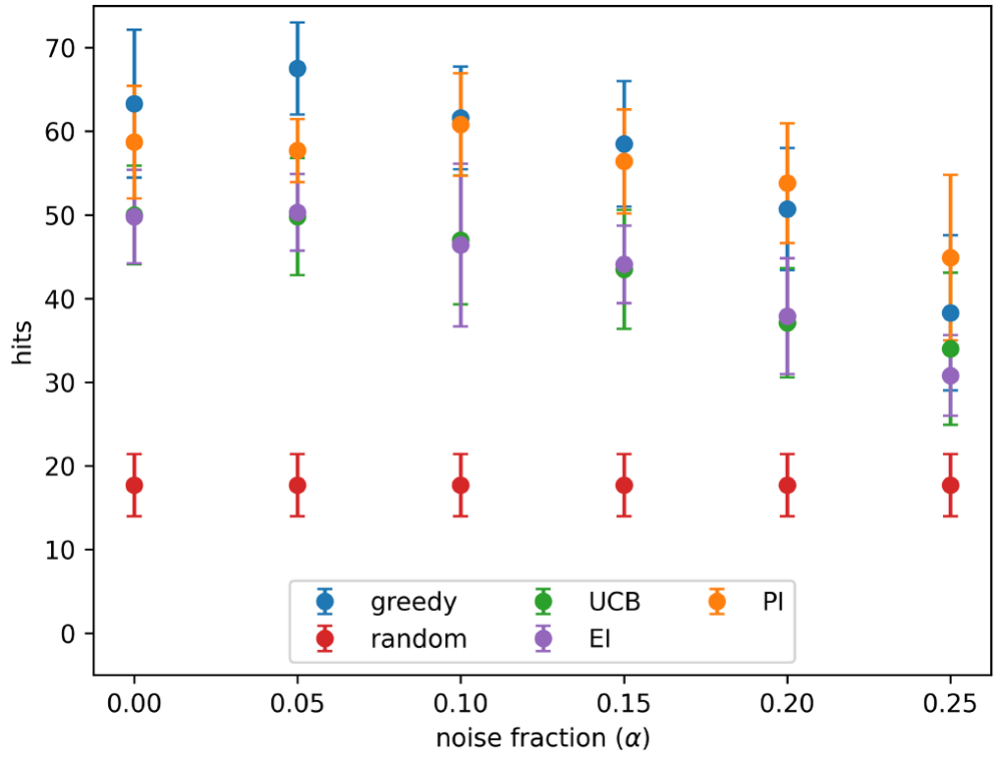
Hits found after 8 active learning batches for each acquisition metric on the PubChem 1893 data set with different levels of noise. Noise added using eq 3 and the indicated values for α. Results show the mean of 10 runs, and the error bars indicate the standard deviation. Acquisition metrics: greedy, random, UCB - upper confidence bound, EI - expected improvement, PI - predicted improvement.

Batched Bayesian optimization was run, both with and without retests, on the two data sets from PubChem. This process was otherwise identical to that used for the simulated data set, except hits were used as defined in the original data set, rather than the top 10% of the data set. Figures 9 and 10 show the number of hits found after 8 active learning batches, at each noise level tested, for the PubChem data sets AID-1347160 and AID-1893, respectively. These results are similar to those for the CHEMBL data set: the greedy and PI acquistion metrics perform the best at all noise levels, UCB and EI are slightly worse, and all metrics outperform random selection.

**Figure 11:**
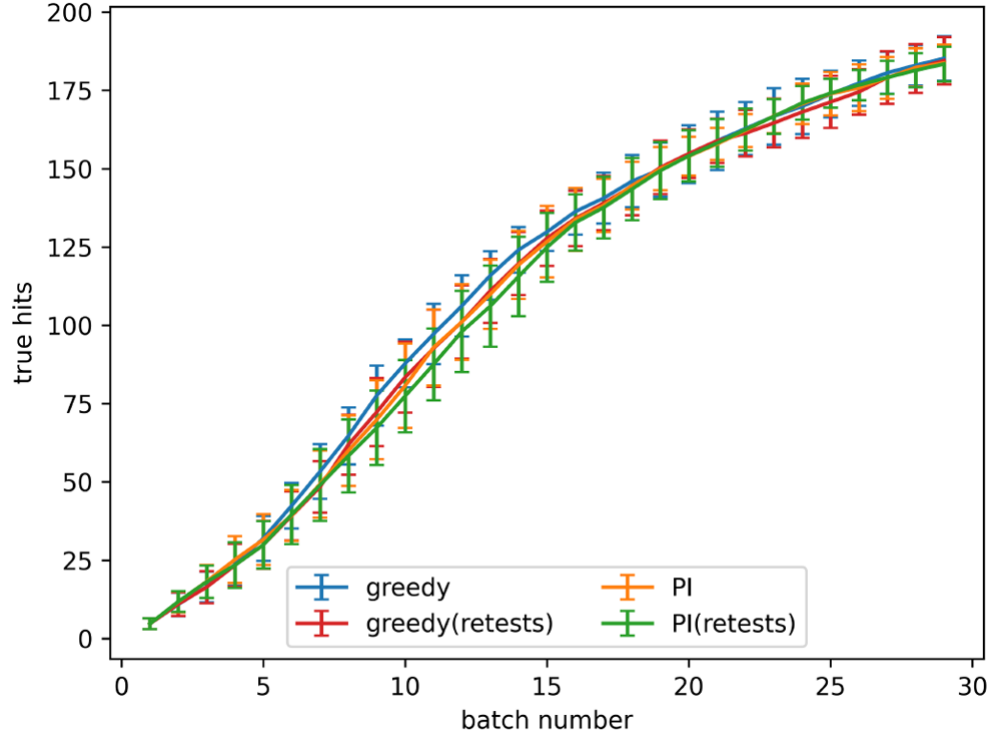
True hits found using active learning, both with and without retests, on the PubChem 1347160 data set for the predicted improvement (PI) and greedy acquisition metrics. Noise added using α = 0.2 in eq 3. The graph shows the mean of 10 runs, and the error bars indicate the standard deviation.

**Figure 12:**
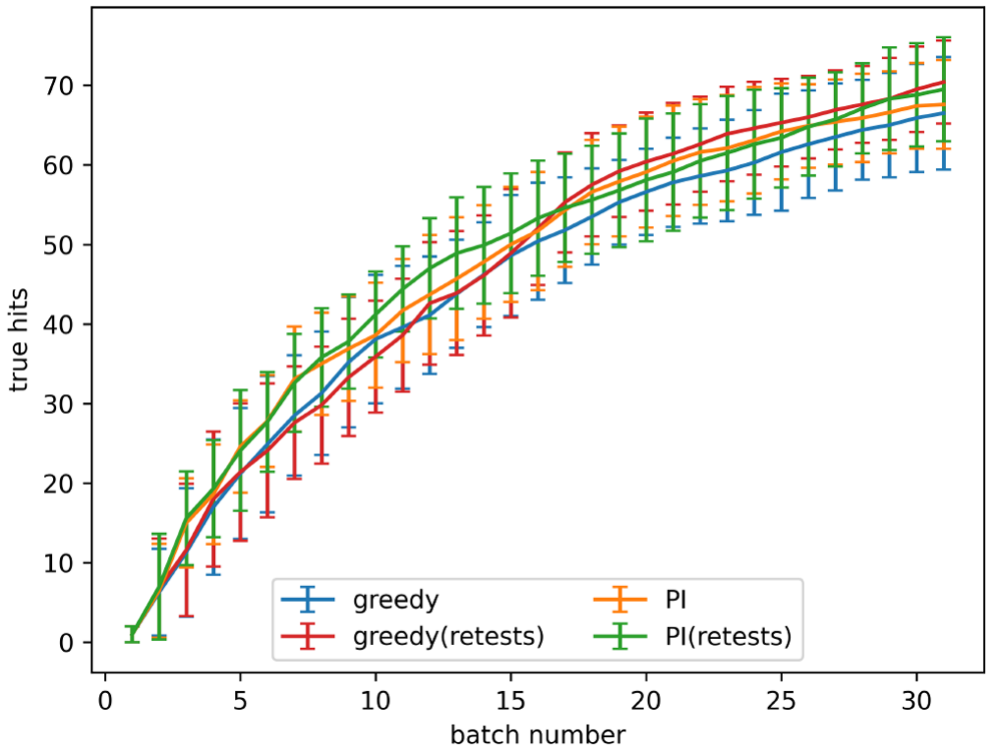
True hits found using active learning, both with and without retests, on the PubChem 1893 data set for the predicted improvement (PI) and greedy acquisition metrics. Noise added using α = 0.2 in eq 3. The graph shows the mean of 10 runs, and the error bars indicate the standard deviation.

Figures 11 and 12 show the acquisition of true hits, both with and without restests, at a noise level of α = 0.2 for the PubChem data sets AID-1347160 and AID-1893, respectively. On both data sets, all of the active learning protocols have a similar performance. Unlike the simulated data set, the number of true hits found is still increasing at the end of the experiment.

**Figure 13:**
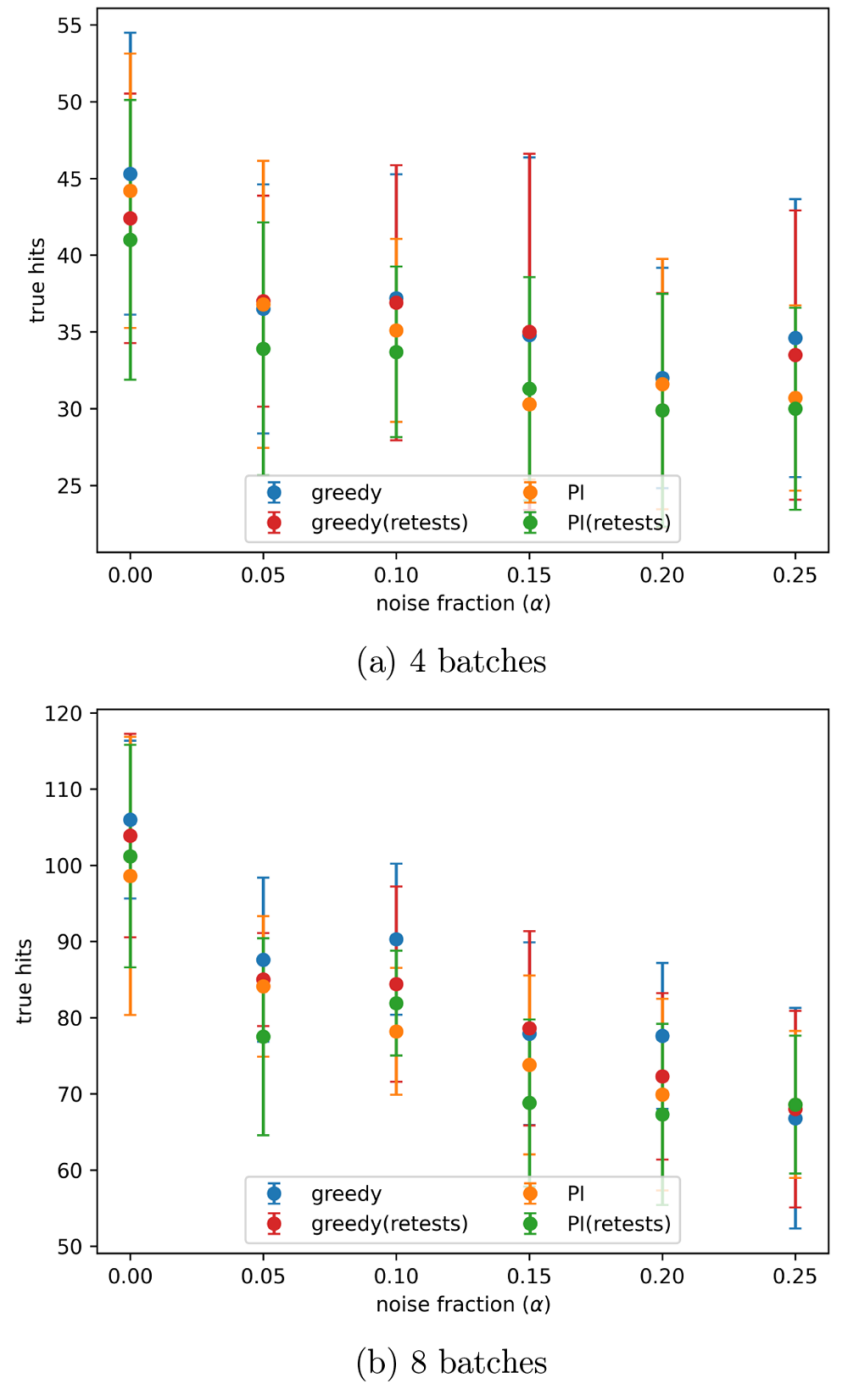
Number of true hits found after the indicated number of active learning batches on the PubChem 1347160 data set, with different levels of artificial noise in the data, both with and without retests. Noise added using the indicated α values in eq 3. Results are the mean of 10 runs with the error bars showing the standard deviation. The acquisition metrics used were greedy and predicted improvement (PI).

**Figure 14:**
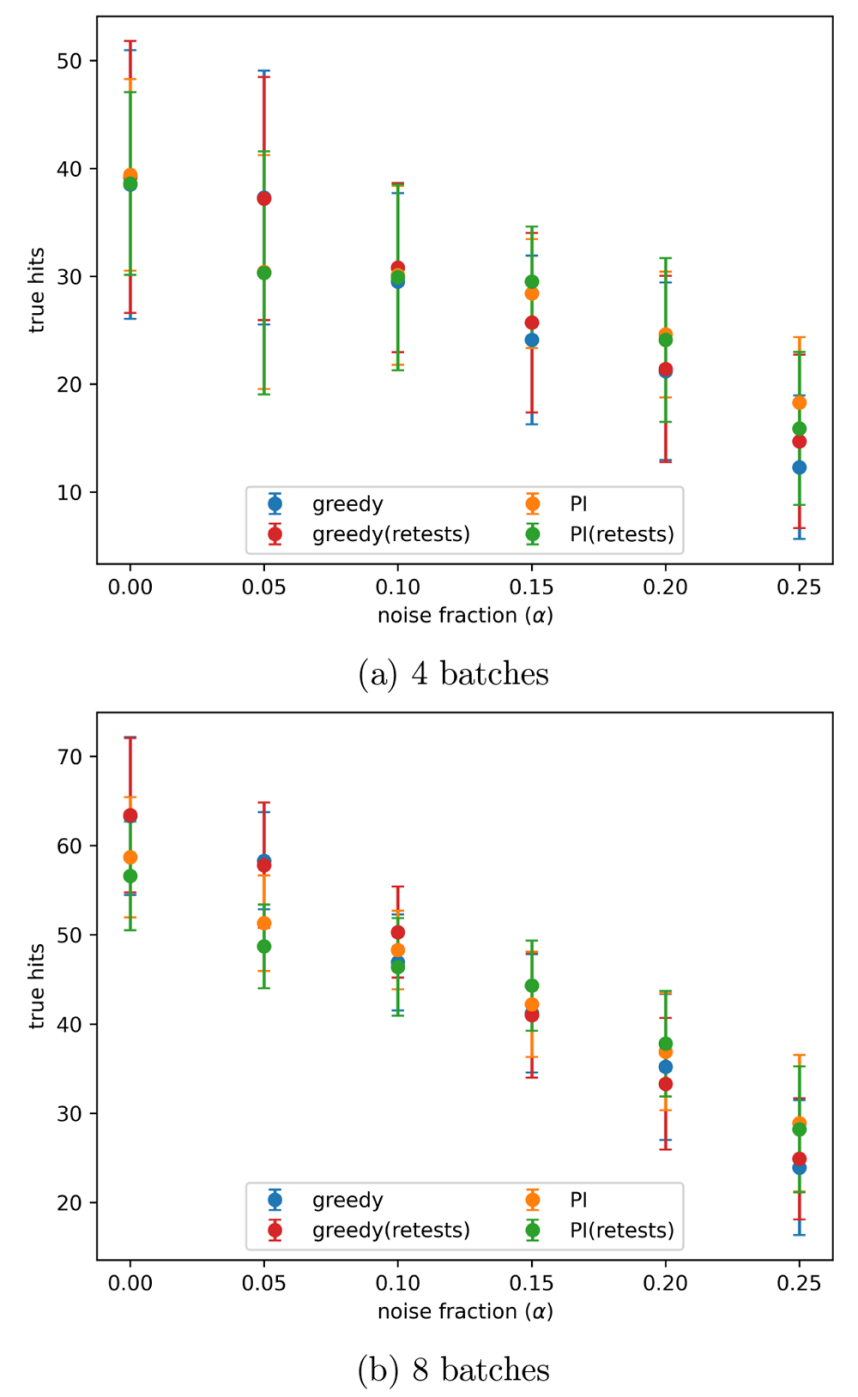
Number of true hits found after the indicated number of active learning batches on the PubChem 1893 data set, with different levels of artificial noise in the data, both with and without retests. Noise added using the indicated α values in eq 3. Results are the mean of 10 runs with the error bars showing the standard deviation. The acquisition metrics used were greedy and predicted improvement (PI).

Figures 13 and 14 show the number of true hits found after 4 and 8 active learning batches, at all noise levels tested, or the PubChem data sets AID-1347160 and AID-1893, respectively. On both of these data sets all of the tested active learning approaches had a similar performance.

## Discussion

### Acquisition Strategy performance

On the simulated data set the greedy and PI acquisition metrics were very effective, finding approximately 5 times as many actives as a random search. On the CHEMBL and PubChem data sets the methods performed worse finding approximately 1.8 times and 2.1 times as many hits, respectively. The suspected reason that the performance is so much worse for these methods is due to differences in the data sets: the simulated data set is an easy function to fit compared to the QSAR problems, and there is less noise present, as the simulated data contains only the artificially added noise (in eq 2 σ_1_^2^ = 0, and noise controlled by changing σ_2_^2^ only), whereas the real data sets have noise present before any additional noise is added (σ_1_^2^ has a fixed value). These data set differences make the real data sets harder to predict using a QSAR model–worse model predictions lead to worse active learning performance. These observations were also reported by Pyzer-Knapp^14^ on a Bayesian optimization study with multiple data sets; the effectiveness of the different acquisition metrics varied between the two different types of data set tested.

The purely exploitative greedy method consistently performs as well or better than the more complex acquisition strategies aiming to balance exploration and exploitation. Similar observations were made by Graff et al. in a structure-based experiment that compared the same acquisition metrics without any artificial noise. They stated that the reason for the poorer performance of the methods that require uncertainty estimates may be that uncertainty quantification in regression models is generally unreliable.^12,26^

The UCB and EI metrics performed very poorly on the simulated data set, but on the real data sets they were similar to the greedy and PI metrics. This could be because the simple function of the simulated data set allowed good predictions to be quickly produced, meaning attempts to explore the data set were not useful, whereas on the real data set further exploration was beneficial, as the models predictions were worse. So, the more exploitative greedy and PI acquisition metrics are much better on the simulated data set, where a very good model can be easily made, and on real data sets the acquisition metrics that perform a more explorative search become more competitive.

**Figure 15:**
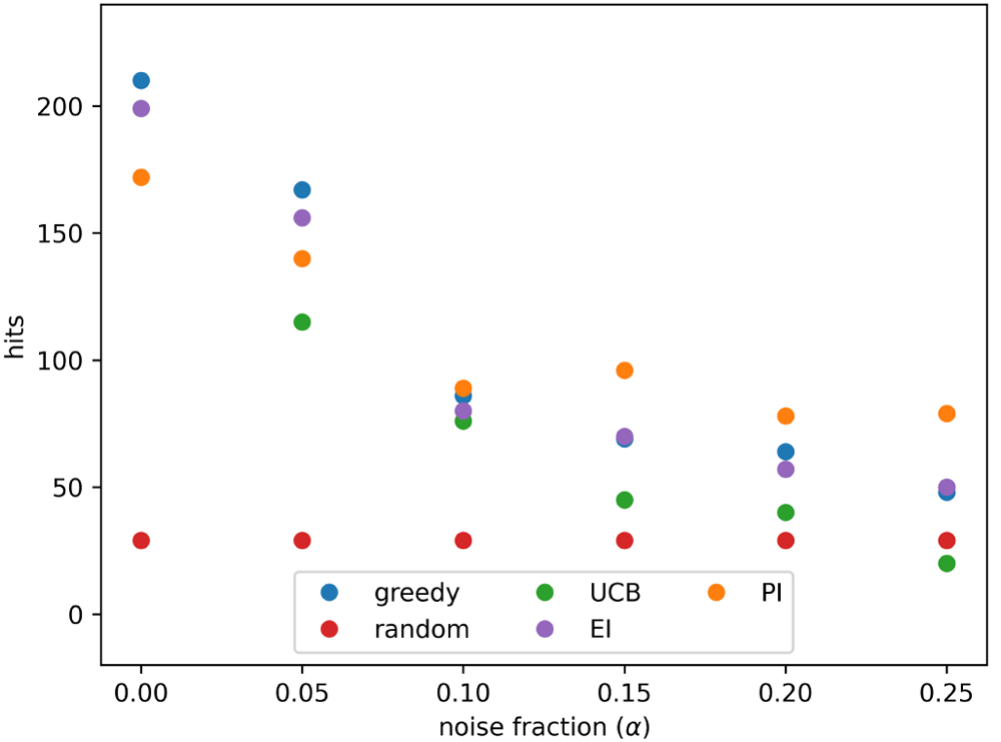
Hits found after 8 active learning batches for each acquisition metric on the new, more complex, simulated data set with different levels of noise. Noise added using eq 3 and the indicated values for α. Acquisition metrics: greedy, random, UCB - upper confidence bound, EI - expected improvement, PI - predicted improvement.

To test this hypothesis the simulated data experiments were repeated with a more complex function. This new data set was produced using the sklearn method make_friedman3, with 5000 samples. The number of hits found after 8 active learning batches for each acquisition metric is shown in Figure 15; these are the same results as were shown in Figure 2 for the original simulated data set. On this more complex data set, the greedy and PI methods perform worse, finding fewer hits. But the EI and UCB methods perform better, showing that a more complex function can allow these explorative methods to perform better.

### Hits Versus True Hits

In a real experimental process, the distinction between hits and true hits is important, as only the true hits will be correctly identified and, so, be considered as prospects for future drugs. The probability of a hit being misidentified decreases as its activity increases (the most active compounds are least likely to be mistaken for nonhits), so the purely exploitative greedy method might be expected to have a better relative performance than the other acquisition metrics on true hits. However, Figure 3 and Figure 7 show the likelihood of a hit being correctly identified is independent of the acquisition function; this is also true for the UCB and EI acquisition metrics, although these results are not shown. This could be because all the acquisition metrics select the compounds with the highest activity, and the difference in performance depends on the compounds near the boundary.

Figure 3 shows that the change in enrichment factor decreases quickly as noise increases. This means that even small amounts of noise present in the data can cause a relatively large fraction of hits to be misidentified and that, as noise increases, the proportion of hits that are misidentified increases more slowly.

### Hit Frequency

**Figure 16:**
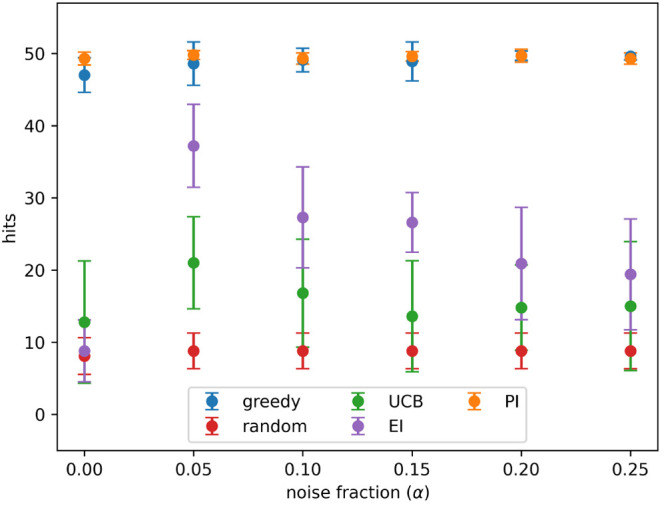
Hits in the top 1% found after 8 active learning batches for each acquisition metric on the simulated data set with different levels of noise. Noise added using eq 3 and the indicated values for α. Results show the mean of 10 runs, and the error bars indicate the standard deviation. Acquisition metrics: greedy, random, UCB - upper confidence bound, EI - expected improvement, PI - predicted improvement.

**Figure 17:**
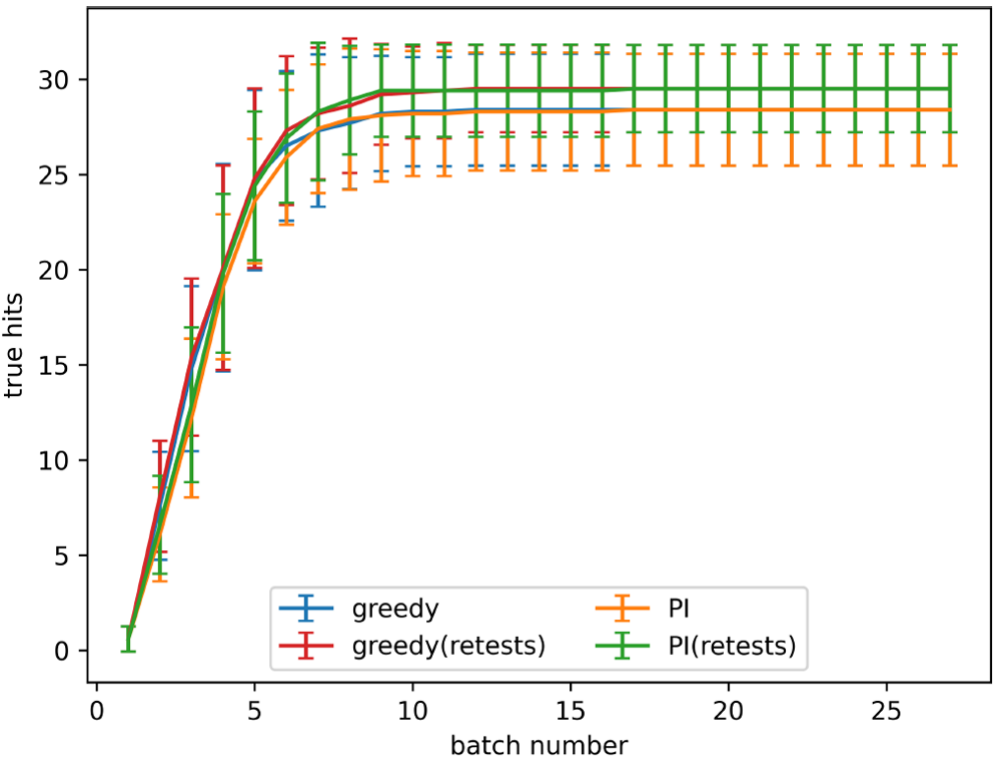
True hits in the top 1% found using active learning, both with and without retests, on the simulated data set for the predicted improvement (PI) and greedy acquisition metrics. Noise added using α = 0.2 in eq 3. The graph shows the mean of 10 runs, and the error bars indicate the standard deviation.

In the simulated data experiment and the CHEMBL experiments hits are taken as the top 10% of the data set. In real drug discovery processes it is likley that active compounds will comprise a smaller fraction of the data set. To investigate the effect of this the simulated experiments were repeated with hits being defined as the top 1%. Figure 16 shows the number of hits found after 8 active learning batches, for each noise level tested, and Figure 17 shows the acquisition of hits at a noise level of α = 0.2.

Figure 16 shows that the change of hit rarity does not effect the relative performance of the acquisition metrics due to its similarity to Figure 2. This is also demonstrated by the results for the PubChem data sets, where the relative performance of the acquisition metrics was similar between the two data sets with hits at approximately 6% for the AID-1347160 (Figure 9) data set and approximatly 2% for the AID-1893 (Figure 10) data set. This means that the frequency of hits does not effect the relative performance of the acquisition metrics.

**Figure 18:**
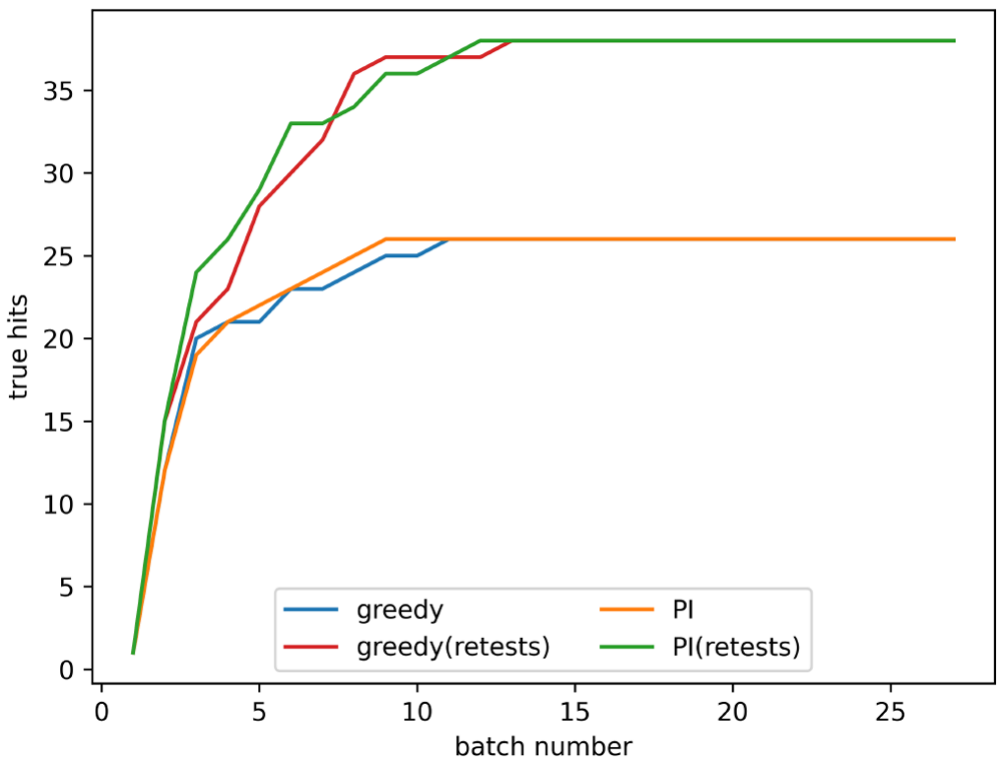
True hits in the top 1% found using active learning, both with and without the alternative retest policy, on the simulated data set for the predicted improvement (PI) and greedy acquisition metrics. Noise added using α = 0.2 in eq 3.

There is a smaller difference between the results with and without retests in Figure 17, when hits are defined as 1%, than in Figure 4, where hits are at 10%. This is because only a very small number of the QSAR model predictions are above the new (1%) activity threshold, so only a very small number of retests occurs, and only a slight improvement is seen in using retests. To demonstrate this a new retest policy was implemented, which retested a molecule if its activity plus a tenth of its prediction uncertainty was above the threshold value. These results are shown in Figure 18. They are much more similar to the results for a 10% hit rarity, and retests have again allowed a much larger number of hits to be correctly identified. This demonstrates that a suitable retest policy must be chosen in conjunction with the model and acquisition metric. This choice will be dependent on the data set and the aims of the active learning process.

### Batch Size

**Figure 19:**
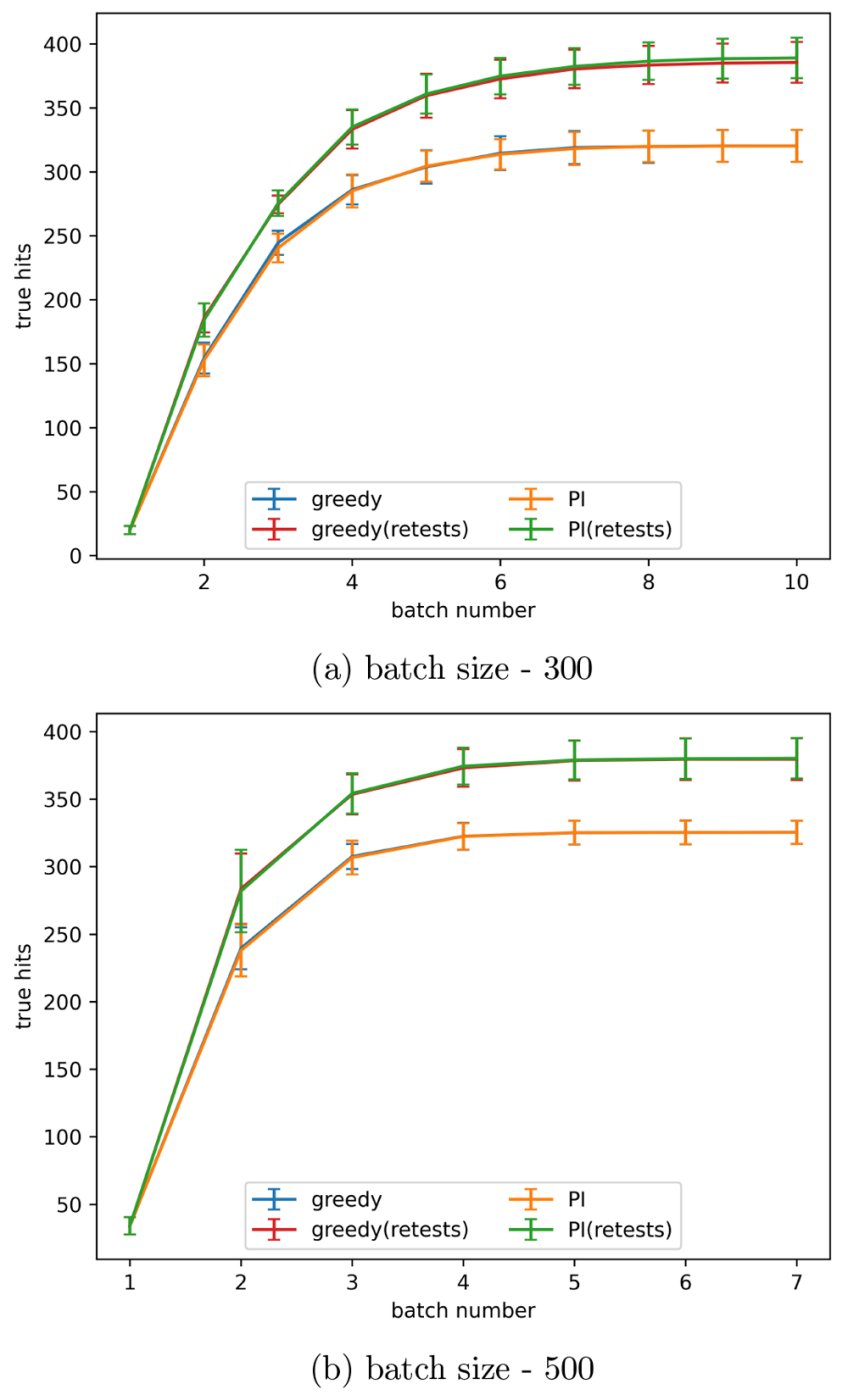
True hits found using active learning, both with and without retests, on the simulated data set for the predicted improvement (PI) and greedy acquisition metrics with the indicated batch size. Noise added using α = 0.2 in eq 3. The graph shows the mean of 10 runs, and the error bars indicate the standard deviation.

In the drug design process the batch size can often be larger than 100. To test the effectiveness of the retest policy the simulated experiment was repeated with batch sizes of 300 and 500. These results are shown in Figure 19. The increasing batch size does not have an effect on the usefulness of the retest policy. This means that retests can be used effectively across a range of batch sizes.

### Usefulness of the Retest Policy

Figure 4 shows that, after a small number of batches, the performance is similar both with and without retests and that, as batch number increases, the retest policy becomes more favorable. The long-term performance with retests finds more hits than without retests; these patterns are also seen in Figure 8. One reason for this is that, at the end of a shorter process, there are still many actives left to be found, making it relatively easy to detect actives by testing on new molecules. At the end of a longer process there are fewer active molecules left to find, making it difficult to detect actives in untested molecules, and so more benefit is gained by retesting molecules.

Using retests also allows for more actives to be possible to identify. For example, at an artificial noise level with α = 0.2, Figure 3 shows that approximately 40% of hits will not be measured as active. So, the maximum number of true hits is about 60% of the total. With retests, if the model performed perfectly, the remaining hits would all be retested, and after 1 retest 60% of them would be correctly classified as true hits. This increases the maximum number of true hits that can be identified from 60% to 84%. Allowing for further retests would increase this amount to
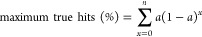
4where *n* is the maximum number of retests allowed, and *a* is the chance a hit is correctly identified as a true hit in the first round. While increasing the number of retests increases the maximum number of hits that can be correctly identified, the error in the QSAR model means that some hits would still be missed. Further retests could cause fewer true hits to be found in the same number of experiments, as fewer unique compounds would be tested due to retests being repeated. The effectiveness of a retest policy, particularly involving multiple retests, will largely depend on the accuracy of the QSAR model.

The retest method found more true hits on both the simulated and CHEMBL data sets, but on the PubChem data sets the performance was very similar both with and without retests. This is due to the data set differences, with actives being more difficult to identify on the PubChem data sets. Figures 11 and 12 show that, on the PubChem data sets, hits are still being identified at the end of the active learning process, whereas in the equivalent figure for the simulated data set (Figure 4) the number of true hits plateaus approximately halfway through the process, showing that all identifiable true hits were found at this point. This difference means that hits are relatively easy to find by doing new experiments on the PubChem data set at all points in the process, reducing any benefit of performing retests. It is expected that, if the active learning process was continued on the PubChem data sets, the retesting methods would eventually become favorable.

Further work could be done on alternative retest methods; these could allow for multiple retests and use the prediction variance, measured activity, and predicted activity to selectively retest compounds. The retests could also be done at the end of the process after a fixed number of batches, potentially giving better performance if the accuracy of the QSAR model improves throughout the process. Additionally, choosing a retest policy depending on the data set to maximize learning performance could be explored.

**Data and software availability**. The code and the data sets used in these experiments are available on github at https://github.com/hugobellamy/JCIM-ALNoise.

## Conclusion

This work demonstrated that batched Bayesian optimization techniques remain effective in noisy environments and that the greedy and PI acquisition metrics preform the best at all noise levels on the tested data sets. Adding noise causes the relative performance of different acquisition metrics to change and makes the absolute performance of the active learning worse. A comparison of results between simulated data and data sets for CHEMBL and PUBCHEM showed that the suitability of different acquisition metrics depends on the data set, surrogate model, and amount of noise. Relatively small amounts of noise can cause many molecules to be misidentified as inactive, and the choice of acquisition metric does not affect the rate at which these molecules are misidentified.

The use of a simple retest policy will increase the amount of correctly identified hits in a fixed number of experiments. In the simulated and CHEMBL data sets the retest policy caused more hits to be found, and on the PubChem data set it resulted in no change to the number of correctly identified hits. This effectiveness of the retest policy was demonstrated with various hit frequencies, and the potential for alternative retest methods, which depend on the hit frequency, was discussed. The retest method was also shown to be applicable for a wide range of batch sizes allowing it to be used flexibly in various active learning procedures.
